# Vitamin D, Chronic Migraine, and Extracranial Pain: Is There a Link? Data From an Observational Study

**DOI:** 10.3389/fneur.2021.651750

**Published:** 2021-05-13

**Authors:** Valentina Rebecchi, Daniela Gallo, Lucia Princiotta Cariddi, Eliana Piantanida, Payam Tabaee Damavandi, Federico Carimati, Marco Gallazzi, Alessandro Clemenzi, Paola Banfi, Elisa Candeloro, Maria Laura Tanda, Marco Mauri, Maurizio Versino

**Affiliations:** ^1^Neurology and Stroke Unit, ASST Sette Laghi di Varese, Varese, Italy; ^2^Endocrine Unit, University of Insubria, Varese, Italy; ^3^Department of Medicine and Surgery, University of Insubria, Varese, Italy; ^4^Clinical and Experimental Medicine and Medical Humanities, Center of Research in Medical Pharmacology, University of Insubria, Varese, Italy; ^5^Department of Medicine and Surgery, University of Milano Bicocca, Monza, Italy; ^6^Department of Biotechnologies and Life Sciences, University of Insubria, Varese, Italy

**Keywords:** chronic migraine, episodic migraine, headache, allodynia, vitamin D

## Abstract

Several studies focused on the role of vitamin D (vitD) in pain chronification. This study focused on vitD level and pain chronification and extension in headache disorders. Eighty patients with primary headache underwent neurological examination, laboratory exams, including serum calcifediol 25(OH)D, and headache features assessment along with three questionnaires investigating depression, anxiety, and allodynia. The 86.8% of the population had migraine (48% episodic and 52% chronic). The 44.1% of patients had extracranial pain, and 47.6% suffered from allodynia. A vitD deficit, namely a serum 25(OH)D level <20 ng/ml, was detectable in 46.1% of the patients, and it occurred more frequently (*p* = 0.009) in patients suffering from chronic migraine (CM)–medication overuse migraine (MOH) (62.9%) than in episodic migraine (EM, 25.7%) or tension-type headache (TTH, 11.4%). The occurrence of extracranial pain and allodynia was higher in the CM-MOH than in the EM and in the TTH groups but was not related to the co-occurrence of vitD deficiency (Fisher's exact test *p* = 0.11 and *p* = 0.32, respectively). Our findings show that 25(OH)D deficit is also related to chronic headache, probably because of vitD anti-inflammatory and tolerogenic properties, reinforcing the idea of a neuroinflammatory mechanism underpinning migraine chronification.

## Introduction

Migraine and tension-type headache (TTH) are common disorders, affecting up to 22 and 78% of the population, respectively (1, 2). Although migraine and TTH are generally episodic and regress taking symptomatic treatments, they may become chronic and necessitate prophylaxis. According to epidemiological studies, each year, 2.5% of episodic migraineurs (EM) progress to chronic migraine (CM), which is characterized by the occurrence of at least 15 migraine attacks per month, at least 8 with migraine features (3–5). CM may favor the development of a wide spectrum of comorbidities, such as psychiatric and sleep disorders, metabolic alterations, along with other forms of pain and medication overuse headache (MOH), diffuse and persistent pain [matching the American College of Rheumatology criteria for fibromyalgia (FMS)], chronic fatigue pain, and myofascial and musculoskeletal pain (1). Accordingly, more than 70% of patients with FMS complain of headache (4–6). The mechanisms leading from EM to CM are not fully understood. Cortical excitability appears to be abnormal in CM patients, compared to EM patients, but this could be a consequence of the disease itself (3). The role and the contribution of inflammation and central sensitization have been considered (7).

Recently, several studies focused on the possible role of micronutrients and especially of vitamin D (vitD) in chronic pain development.

### The Role of Vitamin D in the Pathogenesis of Pain and Headache

Circulating vitD mostly derives from the activation of its endogen precursor (7-dehydrocholesterol) in the epidermis by ultraviolet B radiation, followed by two consecutive hydroxylations (8, 9). In the last decades, a tissue-local production of active vitD was demonstrated (9). Besides the undoubted involvement of vitD in musculoskeletal health, several preclinical studies supported a larger spectrum of activities (8, 10, 11). VitD acts as a developmental neuroactive steroid, influencing various functions of the nervous system and neurotransmitters levels (12, 13). VitD receptors, along with the enzymes involved in vitD synthesis and degradation (25-hydroxylase, 1α-hydroxylase, 24-CYP24A1), are expressed by neurons, astrocytes, and oligodendrocytes distributed in brain regions (14–19), which are therefore able to independently synthetize active vitD and to regulate its local concentrations (12, 16). In the striatum and substantia nigra, vitD seems to be involved in dopamine neurons survival as well as in tonic and phasic dopamine release (20–22). Groves et al. (23) demonstrated that a vitD-deficient diet reduced the expression of enzymes involved in gamma-aminobutyric acid (GABA) synthesis, as confirmed by further experiments (17). Notably, vitD regulates serotonin neurotransmission through the genomic regulation of tryptophan hydroxylase 2 (TPH2) (2, 17). As central nervous system (CNS) cells, most of immune cells express the vitD receptors and the enzymes involved in vitD synthesis and degradation (10). *In vitro* and animal experimental studies could demonstrate that vitD promoted anti-inflammatory and tolerogenic behaviors in both innate and adaptive immune cells, at the expense of proinflammatory subsets (8, 13). Several studies agreed on the existence of a relationship between vitD levels and pain, especially FMS, musculoskeletal pain, and headache (24–27).

The aim of our study was to explore the level of vitD in different kinds of headaches and, more specifically, in relation to pain chronification and the occurrence of chronic extracranial pain and allodynia.

## Methods

Our observational retrospective study aimed at investigating the potential implications of vitD status on headache characteristics and extracranial pain extension.

### Subjects

On the basis of literature data, starting from January 2017 to December 2018, all the patients attended at our center with a diagnosis of primary headache were screened with routine laboratory exams and vitD dosage. Retrospectively, we considered the data from 80 patients who were older than 18 years and who were not pregnant, not suffering from active neoplasia, malabsorption, severe kidney, and cardiovascular disorders, and not taking supplementation with vitD and/or calcium or multivitamin drugs or a treatment for osteoporosis. The patients were divided into six diagnostic subgroups depending on the ICHD-3 criteria (5): EM with and without aura, CM, MOH, TTH, and chronic TTH. For each patient, we acknowledged the following: age, sex, height, weight, body mass index [computed as index and recoded as underweight if <18.49 kg/m^2^, normal if between 18.5 and 24.99 kg/m^2^, overweight if between 25 and 29.99 kg/m^2^, and obese if ≥30 kg/m^2^ (28)], episodes frequency and use of symptomatic drugs that were recorded on specific diaries, the occurrence of chronic extracranial pain (neck, upper and lower back, upper and lower limbs), and allodynia, defined as an Allodynia Symptoms Check (ASC-12) score > 2 with the validated “12-item Allodynia Symptom Check list” (29). We also acknowledged job, physical activity, dairy intolerance, comorbidities, and family history of headache. We administered two questionnaires to each patient: the “Hospital Anxiety and Depression Scale-Anxiety (HADS-A),” which is a well-validated tool to test the presence of depression and anxiety in somatic patients (30–33), and the “Fatigue Severity Scale (FSS),” which investigates fatigue. The HADS-A scale consists of seven items, each with four answer options (scored between 0 and 3 points). The final score defines the absence of anxiety (<7 points), a mild form (8–10 points), a moderate form of anxiety (11–14 points), or a serious disturbance (15–21 points). The FSS questionnaire evaluates the impact of fatigue through nine items, each scored from 1 to 7 points, building a final score between 9 and 63 points (34, 35). The study was conducted in accordance with the “Declaration of Helsinki” and “Good Clinical Practice guidelines.” For this kind of study in which the data are collectable from the clinical records of the patients, we are not required to a have a specific permission from the local ethical committee (Comitato Etico dell'Insubria).

### Blood Samples

Fasting venous blood samples were taken in the morning at least 48 h from the last migraine attack. Serum calcifediol [25(OH)D] concentration is widely considered the most reliable indicator of vitD reserve, reflecting both dietary intake and exposure to UV radiation. Serum [25(OH)D] levels can be assessed by different analytical methods (36); we used a chemiluminescence assay (sensitivity, 1.5 ng/ml; precision interval, 7–11%). The definition of vitD status is still on debate. In this study, according to recent position statement, vitD status was categorized as insufficient for 25(OH)D values <20 ng/ml (37, 38). Intact parathyroid hormone (PTH) was measured by a two-step immunoradiometric assay, sensitivity of 1 pg/ml, normal range of 15–88 pg/ml. Normal values for chemistry and hematology determinations were as follows: calcium (8–10 mg/dl), folates (8.1–45 nmol/L), vitamin B12 (133–675 pmol/L), homocysteine (<12 μmol/L), iron (33–193 μg/dl), and phosphorus (2.5–4.5 mg/dl).

### Statistical Analyses

Demographic and clinical characteristics have been summarized as mean values (±standard deviation) or proportions. The continuous variables were also categorized as normal or abnormal depending on whether the specific value for a given subject fell inside or outside the normal limits. Depending on the type of variable, the analyses were based on either parametric (ANOVA) or non-parametric (chi-square test) methods. The significance value was set at *p* = 0.05.

## Results

The 86.8% of the sample was diagnosed with migraine, episodic (with or without aura) in 48% of the cases and chronic in the remaining 52%. Since CM was complicated by medication overuse (MOH) in 91% of the cases, the CM and MOH groups were lumped together in the CM-MOH group. The remaining 13.2% of the patients suffered from episodic TTH. Thus, a three-category study variable (EM, CM-MOH, and TTH) was taken into consideration for headache diagnosis.

Table 1 displays how the categorial variables were distributed in the three headache diagnostic groups.

**Table 1:** Demographic and clinical data.

	**EM (*n* = 32) 42.1%**	**CM-MOH (*n* = 34) 44.7%**	**TTH (*n* = 10) 13.2%**	**F/chi-square (**χ^2^**); *p***
**Age (years)**	43.1 (15.4)	51.8 (11)	40.1 (14.1)	*F* = 4.9; *p* = 0.01
**MMD [mean (standard deviation)]**	6.1(3.4)	18.9 (4.9)	3.7 (1.5)	*F* = 107.8; *p* <0.001
**MSI [mean (standard deviation)]**	5.6 (3.4)	20.4 (9.9)	3.6 (1.8)	*F* = 48.8; *p* <0.001
**Gender**				
Male	6.2%	8.8%	10%	
Female	93.8%	91.2%	90%	χ^2^ = 0.22; *p* = 0.89
**Work**				
Inside	79.2%	79.4%	88.9%	χ^2^ = 0.46; *p* = 0.70
Outside	20.8%	20.6%	11.1%	
**Sport**				
None	66.7%	71%	50%	χ^2^ = 2.478; *p* = 0.65
Inside	25%	16.1%	12.5%	
Outside	8.3%	12.9%	37.5%	
**BMI**				
<18 kg/m^2^	0%	6.5%	25%	χ^2^ = 5.83; *p* = 0.44
18–24.99 kg/m^2^	75%	63.5%	50%	
>25 kg/m^2^	25%	25%	25%	
**Dairy-free diet**				
Yes	88%	82.4%	87.5%	χ^2^ = 0.774; *p* = 0.679
No	12%	17.6%	12.5%	
**Comorbidities**				
Autoimmune diseases	2.5%	5%	0%	χ^2^ = 12.098; *p* = 0.598
Diabetes mellitus	0%	1.3%	0%	
Fibromyalgia	2.5%	1.3%	3.8%	
Endometriosis and PCOS	3.8%	2.5%	1.3%	
**Menopause**				
Yes	25.8%	51.6%	11.1%	**χ^2^** **=** **7.15;** ***p*** **=** **0.03**
No	74.2%	48.4%	88.9%	
**Extracranial pain**				
Yes	26.9%	64.7%	12.5%	χ^2^ = **12.2;** ***p*** **=** **0.002**
No	73.1%	35.3%	87.5%	
**Allodynia**			35.3%	
Yes	29.2%	66.7%	6.7%	χ^2^ = **10.40;** ***p*** **=** **0.006**
No	70.8%	33.3%	83.3%	
**Prophylactic treatment**				
Yes	42.3%	30.4%	30%	χ^2^ = 0.91; *p* = 0.63
No	57.7%	69.6%	70%	

*EM, episodic migraine; TTH, tension-type headache; CM-MOH, chronic migraine and medication overuse headache; BMI, body mass index; MMD, monthly migraine days; MSI, monthly symptomatic intake; POCS, polycystic ovary syndrome. The statistically significant values are reported in bold*.

The mean age of the CM-MOH group (51.8 years; standard deviation, 11) was slightly but significantly (*F* = 4.87, *p* = 0.01) larger than those of the EM (43.1, 15.4) and of the TTH (40.1, 14.1) groups, and this was reflected by the higher occurrence of menopause in the CM-MOH than in the two other diagnostic groups.

The 44.1% of the cephalalgic patients also had extracranial pain and significantly more (chi-square = 10.4; *p* = 0.006) in the CM-MOH (64.7%) than in the EM (26.9%) and in the TTH (12.5%) groups. The 47.6% of the patients suffered from allodynia, and again, the occurrence in the CM-MOH group was significantly larger (chi-square = 10.4; *p* = 0.006) (66.7%) in the CM-MOH than in the other groups (EM, 29.2%; TTH, 16.7%). On the other hand, the number of subjects with extracranial pain and allodynia was not related to the co-occurrence of vitD deficit as shown in Table 2.

**Table 2:** Association between headache type, pain extension, and vitamin D levels.

	**All patients (*****n*** **=** **76)**		
	**VitD > 20 (*n* = 41)**	**VitD <20 (*n* = 35)**	**F/chi-square (**χ^2^**); *p***
**Age (years)**	**45.5 (15.3)**	**47.9 (12.5)**	***F* = 0.5; *p* = 0.45**
**MMD [mean (standard deviation)]**	**9.4 (7.2)**	**14 (7.9)**	***F* = 7.1; *p* = 0.009**
**MSI [mean (standard deviation)]**	**8.75 (7.2)**	**15.7 (12.2)**	***F* = 9.5; *p* = 0.003**
**Diagnostic group**			
EM	56.1%	25.7%	
CM-MOH	29.3%	62.9%	
TTH	14.6 %	11.4%	χ^2^ = **9.05; p** **=** **0.011**
**Extracranial pain**			
Yes	36.8%	53.3%	χ^2^ = 1.85; p = 0.133
No	63.2%	46.7%	
**Allodynia**			
Yes	42.4%	53.3%	χ^2^ = 0.75; p = 0.27
No	57.6%	46.7%	
**Prophylactic treatment**			
Yes	33.3%	37.9%	χ^2^ = 0.14; p = 0.46
No	66.7%	62.1%	

*EM, episodic migraine; TTH, tension-type headache; CM-MOH, chronic migraine and medication overuse headache; vitD, vitamin D; MMD, monthly migraine days; MSI, monthly symptomatic intake. The statistically significant values are reported in bold*.

Table 3 shows the mean values and the out of normal range values (i.e., percent of subjects falling outside the normal limits) of the continuous variables in the three headache diagnostic groups.

**Table 3:** Main biochemical parameters.

**Variables**	**EM (*****n*** **=** **32)**		**CM-MOH (*****n*** **=** **34)**		**TTH (*****n*** **=** **10)**		**Chi-square; *p***	***F*; *p***
**Variable (abnormal range)**	**OOR**	**Mean ± SD**	**OOR**	**Mean ± SD**	**OOR**	**Mean ± SD**		
**VitD**, ng/ml (<20)	28.1%	19.9 ± 11.4	**64.7%**	20.1 ± 5.9	40%	23.8 ± 17.5	χ^2^ = **9.05;** ***p*** **=** **0.011**	*F* = 0.6; *p* = 0.57
**Ca**, mg/dl (<8.6–>10.2)	3.1%	9.9 ± 0.6	5.9%	9 ± 1.9	0%	9.4 ± 0.2	χ^2^ = 0.80; *p* = 0.7	*F* = 0.31; *p* = 0.74
**P**, mg/dl (<2.5)	3.1%	3.4 ± 0.4	0%	3.3 ± 0.3	0%	3.4 ± 0.3	χ^2^ = 1.39; *p* = 0.5	*F* = 0.3; *p* = 0.74
**PTH**, pg/ml (<15–>88)	8%	33.4 ± 19.6	10.7%	32.5 ± 25.1	0%	37 ± 27.7	χ^2^ = 0.64; *p* = 089	*F* = 0.08; *p* = 0.92
**Folate**, ng/ml (<8.1)	21.4%	15 ± 10.8	33.3%	12.1 ± 5.3	26.8%	17.7 ± 16.2	χ^2^ = 2.06; *p* = 0.7	*F* = 1.14; *p* = 0.33
**Vit B12**, pg/ml (<133)	20.7%	270.1 ± 145.3	6.9%	315.5 ± 141.8	0.0%	385 ± 144.7	χ^2^ = 3.84; *p* = 0.15	*F* = 2.17; *p* = 0.12
**Homocysteine**, μmol/L (>12)	25%	12.8 ± 9.4	47.4%	12.1 ± 3.7	71.4%	14.8 ± 5.8	χ^2^ = 5.1; *p* = 0.08	*F* = 0.31; *p* = 0.73
**Fe**, μmol/dl (<33)	15.4%	97.9 ± 43.3	19.2%	76.2 ± 41.8	0.0%	97.7 ± 22.3	χ^2^ = 1.38; *p* = 0.5	*F* = 1.98; *p* = 0.15

*EM, episodic migraine; CM-MOH, chronic migraine and medication overuse headache; TTH, tension-type headache; Ca, calcium; Fe, iron; OOR, out of range; P, phosphate; VitB12, Vitamin B12; vitD, Vitamin D; OOR, out of range. The statistically significant values are reported in bold*.

The analyses of variance invariably proved that the mean values of the three groups were not statistically different. As for the occurrence of out of range values, the only significant comparison involved the vitD deficit that was larger in the CM-MOH than in the other two groups.

The season of enrolment (Table 4) did not significantly influence 25(OH)D concentrations. In detail, almost half of the patients suffering from vitD deficit (45.8%) were enrolled during spring–summer season. Moreover, the mean value of vitD measured during the spring (20.09 pg/ml) and the one measured during autumn (20.7 pg/ml) were not statistically different (*F* = 0.69; *p* = 0.79).

**Table 4:** Seasons of enrolment in the whole sample divided by vitamin D levels and headache categories.

**Season**	**All (*n* = 76)**	**VitD > 20 ng/ml (*n* = 41)**	**VitD <20 ng/ml (*n* = 35)**	**Chi-square; *p***	**EM (*n* = 32)**	**CM-M0H (*n* = 34)**	**TTH (*n* = 10)**	**Chi-square; *p***
Aut–Wint	42.1%	43.9%	40%)	χ^2^ = 0.118; *p* = 0.46	40.6%	38.2%	60%	χ^2^ = 1.55; *p* = 0.46
Spring–Sum	57.9%	56.1%	60%		59.4%	61.8%	40%	

*Aut–Wint, Autumn–Winter; Spring–Sum, Spring–Summer; EM, episodic migraine; CM-MOH, chronic migraine and medication overuse headache; TTH, tension-type headache; vitD, vitamin D*.

*Data are reported as number (frequency)*.

Regarding the analyses of questionnaires, the mean FFS scale final score in the whole sample was 36 points. The subgroups suffering from EM and CM-MOH got higher scores at FFS (respectively, 43 ± 15.1 and 41 ± 14.7 points) compared to the TTH group (29.3 ± 14.4), but these differences were not statistically significant (*F* = 0.98; *p* = 0.38). According to the results of the HADS-A questionnaire, the three groups suffered from a mild state of anxiety (respectively, EM 10.5 ± 4.7, CM-MOH 8.9 ± 4.5, and TTH 10 ± 2.6 points), again not statistically significant (*F* = 0.44, *p* = 0.65).

We compared the mean values and the occurrence of an abnormal values using the occurrence of vitD deficit as the explanatory variable, but all these comparisons did not prove to be significant.

The BMI group that the subjects belonged to was independent from the diagnostic group; moreover, if BMI was considered as a continuous variable, the BMI mean values did not depend on the occurrence of vitD deficit (*F* = 1.3; *p* = 0.252) nor on the diagnostic group (*F* = 0.3; *p* = 0.74). The level of vitD and BMI were not significantly correlated (*r* = −0.22; *p* = 0.096).

Finally, the ongoing pharmacological treatment (both prophylactic and abortive) was not different depending on the headache diagnostic group and on the occurrence of vitD deficit.

## Discussion

The main result of our study was that the occurrence of vitD deficiency was more frequent in the CM-MOH than in the EM and TTH groups. A similar results was detectable both for monthly migraine days and the migraine symptomatic intake that were larger in the vitD-deficit group, but these results are explicable because the patients with a high frequency of attack and of symptomatic intake belong to the CM-MOH group; moreover, pain killers are not listed among the causes for vitD deficit.

This result, neither influenced by the season of evaluation nor by the patient lifestyle or headache treatment, supported the hypothesis of a relationship between vitD status and the diagnosis of CM-MOH. The vitD deficiency was not significantly associated with any of the other variables that we considered, not to clinical features such as extracranial pain, allodynia, and prophylactic treatment, nor to biological parameters. None of the other micronutrients tested proved to be different in the three diagnostic groups.

Our findings are in keeping with recent data that showed that poor vitD status was correlated to chronic pain (39) and with a high frequency of migraine episodes (40), but it is worth pointing out that the absence of correlation between vitD deficit and extracranial pain or allodynia, despite that they frequently occur in MOH patients, suggests that they do not stem from the same pathophysiological mechanism of chronification. The mechanisms underpinning migraine chronicity are not fully clarified, but they seem to be connected to a sensitization process acting at a peripheral level first and at a central level afterwards (3). Central sensitization, driven by increased neuronal responsiveness and neuroinflammation, might perpetuate pain, even in the absence of any trigger (41). More recently, the role of the immune system along with neuropeptides release and blood flow modifications was also highlighted in the mechanism of chronification (41–43).

VitD mitigates inflammation by the reduction in proinflammatory mediators [such as interferon (IFN)-gamma; interleukin (IL)-1 beta, IL-6, and IL-17; tumor necrosis factor alpha (TNF-alpha)], favoring expansion of anti-inflammatory molecules (IL-4, IL-5, IL-10) (44). Notably, vitD reduces levels of the “nuclear factor kappa-light-chain-enhancer of activated B cells” (NF-kB), which is a pivotal agent in inflammation (45). Thus, vitD is supposed to counteract neuroinflammation as well (46, 47).

An intervention study demonstrated that the reduction in proinflammatory cytokines by vitD supplementation was correlated to the improvement of musculoskeletal pain (48).

The regulation of oxidative balance is another potential mechanism of vitD anti-inflammatory action (17, 49, 50). As demonstrated by several trials, neurogenic inflammation in chronic migraine is mediated by oxygen free radicals (ROS) and nitric oxide (NO) concentrations (51–54). Togha et al. (52) demonstrated that chronic migraineurs had lower total antioxidant non-enzymatic capacity and higher ROS levels than EM patients (52). Physiological vitD level decreases intracellular oxidative stress-related activities, upregulating the expression of several genes implicated in mitochondrial activity, defense against oxidative burst and aging, and particularly of the nuclear factor erythroid-2(Nf-E2)-related factor and of Klotho (45, 55–59).

Obesity is a proinflammatory condition and bears the potential to intensify neurovascular inflammation. Previous reports observed that high BMI was correlated to severity and frequency of migraine episodes (60). In our study, this association was not confirmed, but the small number of overweight people (none suffering from obesity) could be a possible explanation. Moreover, in our patients, the BMI and vitD deficiency were no related.

### Limitations

Our study has two main limitations. It is a retrospective not interventional study, and the sample size is not so large. The availability of many parameters for each patient partly compensate for the first limitation, but, on the other hand, their usability is still statistically limited by the sample size.

## Conclusion

Although, preliminary, our results show an association between CM-MOH and vitD deficit, probably reflecting the vitD anti-inflammatory and tolerogenic properties, we did not find the same relationship between vitD deficit and extracranial pain and allodynia, thus suggesting that their pathophysiological mechanism is not exactly the same of pain chronification in CM-MOH.

## Data Availability Statement

The raw data supporting the conclusions of this article will be made available by the authors, without undue reservation.

## Ethics Statement

Ethical review and approval was not required for the study on human participants in accordance with the local legislation and institutional requirements. Written informed consent for participation was not required for this study in accordance with the national legislation and the institutional requirements.

## Author Contributions

VR, LP, DG, and PT collected the data and performed the neurological and endocrinological assessment. MM and MV did the statistical analyses. All authors contributed in interpreting the data and writing the manuscript, read and approved the final manuscript, and have agreed both to be personally accountable for the author's own contributions and to ensure that questions related to the accuracy or integrity of any part of the work are appropriately investigated, resolved, and the resolution documented in the literature.

## Conflict of Interest

The authors declare that the research was conducted in the absence of any commercial or financial relationships that could be construed as a potential conflict of interest.

## Abbreviations

CM: chronic migraine
vitD: vitamin D
EM: episodic migraine
TTH: tension-type headache
MOH: medication overuse headache
IFN: interferon
IL: interleukin
NF-kB: nuclear factor kappa-light-chain-enhancer of active B cells
NO: nitric oxide
ROS: oxygen free radicals
TNF: tumor necrosis factor
FMS: fibromyalgia
BMI: body mass index
HADS-A: Hospital Anxiety and Depression Scale-Anxiety
FSS: Fatigue Severity Scale.
